# Analysis of Interfacial Properties and Mechanical Mechanisms of Expansion-Type Bolts

**DOI:** 10.3390/ma19204264

**Published:** 2026-10-09

**Authors:** Yixiao Wang, Wei Zhao, Pengyu Zhu, Yuliang Lin

**Affiliations:** 1 National Engineering Research Center of High Speed Railway Construction Technology, Central South University, Changsha 410075, China; 2 China Railway No. 2 Engineering Group Co., Ltd., Chengdu 610031, China

**Keywords:** pull-out test, similar model, anchorage mechanism, expansion-type rock bolt, pull-out strength

## Abstract

As mining operations extend to greater depths, the high in-situ stresses and intense disturbances associated with deep mining present significant challenges for mining operations, including fractured rock masses, poor stability, susceptibility to water-induced softening and deformation, and difficulties in ground support. As one of the most critical support components in tunnels and mine roadways, the anchorage mechanism of bolts at the bolt-surrounding rock interface in deep fractured surrounding rock remains unclear. To gain deeper insight into the anchorage mechanism and failure evolution of the bolt-surrounding rock interface under deep surrounding rock conditions, this study conducts similar bolt pull-out tests to investigate the anchorage performance of the bolt-surrounding rock interface during pull-out under multiple experimental factors, and analyzes the mechanical properties, response stages, and failure characteristics of the anchorage interface using multiple evaluation indices. The results show that: (1) the anchorage length, the confining pressure, and the material strength act on the anchorage performance through distinct mechanisms; (2) the introduction of expansion points on the bolt surface alters the interfacial load-transfer path and markedly improves the anchorage performance, albeit with a marginal effect; (3) based on the measured load-slip responses, the pull-out process is interpreted as six consecutive stages, whose characteristics differ notably between conventional and expansion-type bolts.

## 1. Introduction

Mining production, especially rare metal mining, is of major strategic importance to China. Mining at ever-greater depths is likewise underway worldwide, such as at the gold and platinum mines in South Africa and the copper mines in South America [1,2]. However, after years of continuous extraction, most mines have now entered the deep mining stage. In deep geological environments characterized by the coexistence of high in-situ stresses and complex joint-fracture structures, the surrounding rock exhibits severe fracturing [3,4], insufficient self-stabilization capacity [5,6], susceptibility to water-induced softening, and large deformation [7,8], imposing extremely high demands on anchoring and support in mining engineering. As one of the most critical support components in tunnels and mine roadways, bolts directly control the deformation and failure of the surrounding rock by applying radial confinement around the roadway periphery and forming a reinforced load-bearing arch [9]. Deep roadway support mainly consists of bolting alone or bolting combined with other support forms, which has given rise to numerous improved bolts and corresponding support structures suitable for deep roadways. Among them, some bolts exploit the expansive action generated by grouting to form multiple expansion points at the bolt-surrounding rock interface, offering significant advantages in pull-out resistance compared with conventional bolts, and have therefore attracted increasingly widespread attention [10,11,12,13].

However, the failure of the above anchorage systems in many engineering cases does not originate from insufficient strength of the bolt body itself [14], but from anchorage failure at the bolt-surrounding rock interface, whereby the bolt is pulled out completely long before its material ultimate strength is reached. The characteristic of the bolt-surrounding rock interface failing first means that the mechanical potential of the bolt material is not fully utilized, and the safety reserve of the anchorage system is severely weakened [15]. Under the high in-situ stress conditions of deep surrounding rock, the failure process of the interface anchorage is more complex [16,17], and the coupling of multiple factors, including surrounding rock properties and mining-induced disturbances, causes the failure of the deep anchorage interface to exhibit characteristics distinctly different from those in shallow conditions [18,19,20]. Therefore, an in-depth understanding of the anchorage mechanism and failure evolution of the bolt-surrounding rock interface under deep surrounding rock conditions is of great practical significance for improving the reliability of support design and the durability of anchorage systems in deep mining engineering.

The pull-out test is widely regarded as the most direct and reliable test method for evaluating the mechanical properties of the bolt–surrounding rock interface, and its results provide a critical basis for the establishment of anchorage theoretical models, either analytical formulations [21] or discrete-element numerical simulations [22], and for the calibration of interface parameters in numerical models [23]. Compared with in-situ pull-out tests, laboratory pull-out tests enable independent control of different key influencing factors, making multi-factor coupled analysis possible. Depending on the research purpose and the simulation object, laboratory pull-out tests can be divided into full-scale pull-out tests, similar model pull-out tests, sleeve pull-out tests, and other common forms. Research systems such as electro-hydraulic servo closed-loop loading systems, fiber Bragg grating (FBG) demodulation systems, three-dimensional digital image correlation (DIC) full-field strain measurement systems, and acoustic emission (AE) damage localization systems [24,25] are used to analyze factors such as the anchorage mode, loading path, boundary conditions, and surrounding rock materials, thereby effectively revealing the interfacial mechanical behavior and failure mechanism under the corresponding working conditions [26,27,28].

Considering that similar model tests have the advantages of controllable boundary conditions, independently adjustable variables, high repeatability, and standardized specimen preparation [29], and given the objective constraint that in-situ pull-out tests are difficult to carry out in deep surrounding rock field environments, this study adopts the similar model test method based on previous research results [30,31,32,33,34] to further reveal the mechanical characteristics and failure mechanism of the bolt-surrounding rock anchorage interface in deep field environments. Multiple factors, including anchorage length, confining pressure, material mix proportion, and bolt type, are set, and multi-dimensional evaluation indices are selected for analysis [35,36]. The research results have theoretical reference value and engineering guidance significance for the optimization design of anchorage support system parameters and the assessment and control of anchorage interface failure risks in deep underground engineering.

Expansion-type bolts are used in practice to improve the pull-out resistance in weak or fractured ground, where conventional fully grouted bolts may fail at the bolt–rock interface. Existing studies have mainly examined the load transfer of conventional fully grouted bolts, whereas the anchorage behaviour of expansion-type bolts has only recently been addressed. The combined effects of anchorage length, confining pressure, and surrounding-rock strength on such bolts have not been investigated systematically, and the failure stages of the interface during pull-out remain poorly quantified. The novelty of the present study lies in the simultaneous evaluation of anchorage length, confining pressure, material strength, and expansion-point configuration within a single similar-material framework, together with a multi-indicator characterization of the interfacial behaviour. In deep mines, the high in-situ stress, fractured rock masses, and water-induced softening impose particular demands on the post-peak and long-term performance of anchorage systems, which motivates the present study. The main objectives are therefore to (i) clarify how anchorage length, confining pressure, material strength, and expansion-point configuration govern the anchorage performance, and (ii) identify and characterize the failure stages of the bolt–surrounding rock interface during pull-out.

The remainder of this paper is organized as follows. Section 2 describes the experimental program, including the model bolt and expansion-point fabrication, the similar materials and mix proportions, the test apparatus, and the test procedure. Section 3 presents the results of the multi-indicator analysis under the different test variables. Section 4 discusses the pull-out failure stages and the bolt deformation and failure. Section 5 summarizes the main conclusions.

## 2. Experimental Program

### 2.1. Expansion-Type Rock Bolts

The aim of the pull-out tests is to characterize the anchorage performance of the bolt–surrounding rock interface under deep-ground conditions. Expansion-type rock bolts are bolts on which one or several enlarged sections (expansion points) are formed along the anchorage length, typically by grout bulbs created through staged or pressurized grouting [37,38]; they are used in practice to increase the pull-out resistance in weak or fractured ground. A commercial expansion-type bolt could not be tested directly at the present model scale because its geometry cannot be scaled down while preserving the grouting details; therefore, a model bolt with geometrically similar expansion points was fabricated as described below.

**Figure 1:**
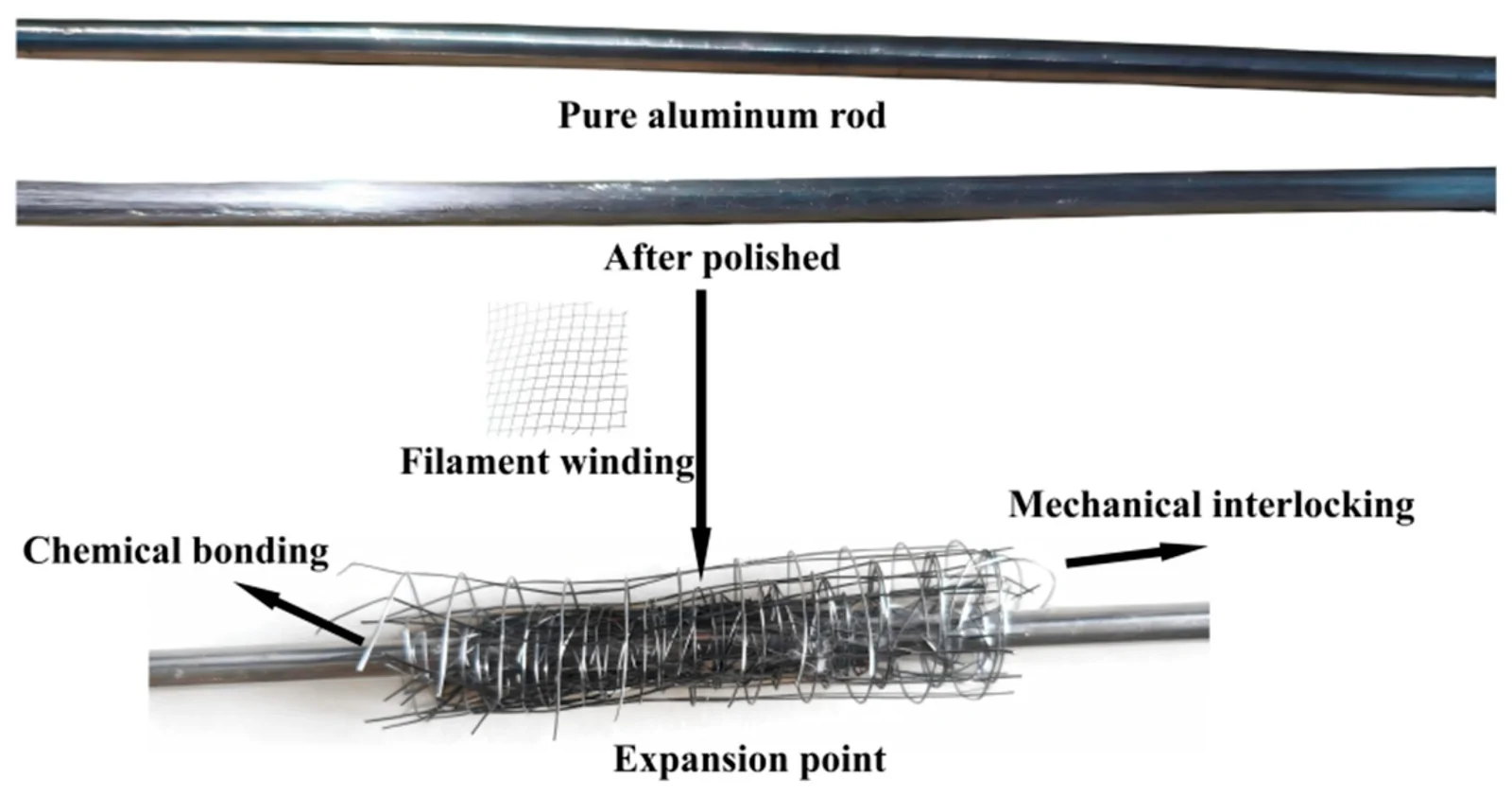
Expansion-type rock bolts.

The rod is made of 1050 commercially pure aluminium (≥99.6% Al) with a diameter of 5 mm and a nominal yield strength of greater than 20 MPa; under the geometric similarity ratio of 1:5, it represents a prototype bolt with a diameter of 25 mm whose scaled strength corresponds to that of common rock-bolt steels. This rod was selected as the model bolt because its low yield strength preserves the strength contrast between the bolt and the surrounding rock at the model scale, so that the interfacial failure, rather than bolt fracture, governs the pull-out response—as in the field. The rod was abraded with sandpaper before the tests. For the expansion-type rock bolt, a wire-wound resin-encapsulated structure was adopted in this test to simulate the expansion point. The specific fabrication process is as follows: a steel wire mesh with a mesh size of 6 mm and a wire diameter of 0.3 mm was wound around the bolt to the specified size to form the expansion point, and a single-component ethyl α-cyanoacrylate adhesive together with a single iron wire was used to fix the expansion point to the bolt. During the actual test, the above expansion points could effectively contact the epoxy resin serving as the anchoring agent, forming a wire-wound resin-encapsulated composite expansion body, thereby effectively simulating the grouting operation and composite anchorage behavior of the expansion-type rock bolt. No obvious slip of the expansion points was observed during the pull-out process, further demonstrating the rationality of the similar expansion point fabrication. The specific configuration of the similar bolt materials is shown in Figure 1.

The fabricated expansion points reproduce the essential geometric and bonding features of grout bulbs—an enlarged diameter over a finite length along the anchorage section—and the epoxy casting ensures a monolithic connection between the expansion point and the surrounding matrix, which is the primary load-transfer mechanism of grouted expansion bolts. The validation is limited to this geometric and bonding equivalence; the grouting pressure itself is not reproduced at the model scale.

### 2.2. Materials and Mix Proportions

The meso-structure of the surrounding rock similar material in this test consists of three categories of constituents: skeleton grains, binding materials, and other additives. Owing to its relatively stable physicochemical properties and mineralogical similarity to natural rocks, quartz sand has been widely used as the skeleton grain material for preparing similar materials of soft rock. The quartz sand used in this test has a SiO_2_ content of 99%, effectively reducing the interference of impurities on the simulation of rock mass mechanical properties. Quartz sand particles of different mesh sizes—20–40 mesh, 40–100 mesh, 100–200 mesh, and 300–400 mesh—were mixed in a component ratio of 2:5:2:1 to form the skeleton grains of the surrounding rock similar material in this test. The binding material is the key constituent that maintains contact between and transfers stress among the skeleton grains of the fractured rock mass. In this test, β-hemihydrate gypsum was selected as the primary cementitious material, and cement with a strength grade of C30 was used as the secondary cementitious material. Among them, β-hemihydrate gypsum has rapid-setting and micro-expansion characteristics, enabling rapid solidification and filling of intergranular voids in the early stage of specimen forming, providing early consolidation and densification of the skeleton grains and constituting the main bonding strength of the binding material; the incorporation of cement aims to moderately regulate the hydration reaction process and finely tune the later-stage stiffness of the cementitious system, so as to more accurately match the mechanical response characteristics of deep fractured rock masses after localized compaction. Other additives mainly consist of water, which is used to bond the materials together, together with a trace amount of citric acid as the retarder for the β-hemihydrate gypsum.

To investigate the interfacial anchorage mechanical properties under different surrounding rock similar materials, five types of surrounding rock similar materials with different mix proportions were fabricated in this test, and the mix proportion of each material is shown in Table 1. The specimens were mixed and molded according to the material proportions in the table, placed into molds for pressure compaction, and tested after 24 h of curing.

In this study, a commercial E44 epoxy resin (component A) with a polyamide curing agent (component B) was selected as the anchoring agent. To ensure complete curing and full-strength development under the aforementioned curing conditions, components A and B were mixed at a weight ratio of 1:1 before use. According to the manufacturer’s data, the mixture has a viscosity of 5000–6000 mPas at 25 °C, an operating time of about 30 min per 100 g, and a steel–steel shear strength of not less than 8 MPa, which is comparable to resin grouts used in practice. The specimens were cured for 24 h at laboratory temperature, which corresponds to the complete curing time of the resin specified by the manufacturer (initial curing 4–6 h and complete curing 24 h at 25 °C), ensuring stable mechanical properties before testing.

### 2.3. Experimental Setup and Procedure

The laboratory pull-out test apparatus for the bolt-surrounding rock similar material mainly consists of a pull-out device, a pressurization device, and various monitoring devices. The monitoring devices used in the test include an MRHZ pressure sensor (Anhui Mirui Instrument Co., Ltd., Bengbu, China) with an accuracy of 0.1 N; an HG-C1700 displacement sensor (Shanghai Kedi Industrial Co., Ltd., Shanghai, China) with an accuracy of 0.1 mm; and an SZYB-Ea miniature earth pressure cell (Hunan Sanzhi Yingke Measurement and Control Technology Co., Ltd., Changsha, China) used to ensure the consistency of the test conditions. A DY3818Y 16-channel static strain acquisition instrument (Jiangsu Donghua Testing Technology Co., Ltd., Taizhou, China) was used to read part of the monitoring data. The surrounding-rock model specimens were prepared with overall dimensions of 600 mm × 200 mm × 200 mm (length × width × height), and the model bolt was a 5 mm-diameter aluminium rod with a total length of 1000 mm, of which the anchorage length ranged from 10 to 60 cm according to the test groups. Strain gauges were bonded to each bolt at intervals of approximately 10 cm, with five gauges on each bolt, so that the deformation of the bolt could be recorded along its length during pull-out.

**Figure 2:**
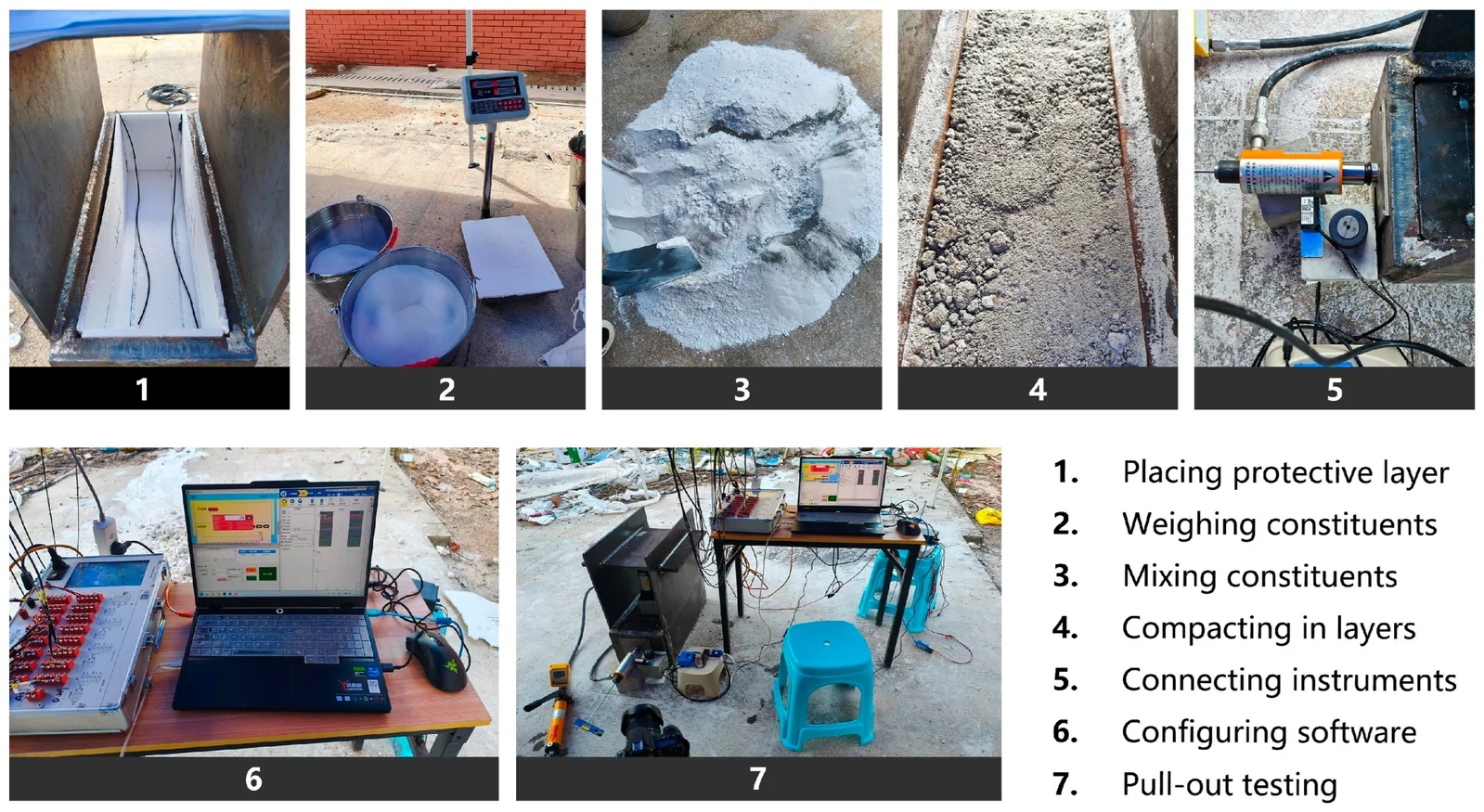
Experimental procedure.

The complete procedure for each group of tests is shown in Figure 2. Before the test starts, a protective material is laid on the inner wall of the mold (Step 1). The materials are then prepared and the specimens molded according to the mix proportions in Section 2.2 (Steps 2–4), in Step 4, the bolt of the corresponding group is embedded and the anchoring agent is added. Next, the experimental instruments are connected and tested according to the test apparatus described above (Steps 5–6). Finally, the test is conducted (Step 7), and the above procedure is repeated until the entire test program is completed.

## 3. Result Analysis

### 3.1. Anchorage Performance Indicators

Pull-out tests on similar bolt materials usually characterize the slip-deformation relationship of the bolt similar material in the similar surrounding rock matrix through the load-slip curve. Drawing comprehensively on relevant studies, this paper selects the extreme load (*F_max_*), the strength utilization ratio (*ρ_f_*), the pull-out energy (*W_P_*), the specific pull-out energy (${\bar{W}}_{P}$), the maximum pull-out stress (*σ_f_*_,*max*_), the average bond strength (*τ_av_*), and the equivalent bond strength (*τ_eq_*) to evaluate the interfacial anchorage performance between the bolt similar material and the similar surrounding rock matrix.

The extreme load (*F_max_*), the maximum pull-out stress (*σ_f_*_,*max*_), and the strength utilization ratio (*ρ_f_*) are key indices characterizing the mechanical behavior of the pull-out material. The extreme load (*F_max_*) refers to the peak interaction force between the bolt and the rock mass during the pull-out process. The maximum pull-out stress (*σ_f_*_,*max*_) reflects the maximum tensile stress sustained by the bolt during pull-out, expressed as:

(1) $$\sigma_{f,\max}=\frac{4F_{\max}}{\pi {D_{F}}^{2}}$$

where *F_max_* denotes the extreme load, and *D_F_* denotes the bolt diameter.

The strength utilization ratio (*ρ_f_*) is used to characterize the inherent strength reserve of the bolt during the pull-out process. A higher value indicates a greater tendency for the bolt to fail by tensile fracture rather than pull-out failure, expressed as:

(2) $$\rho_{f}=\frac{\sigma_{f,\max}}{f_{\mathrm{tf}}}$$

where *f_tf_* denotes the ultimate tensile strength of the aluminium rod, determined by uniaxial tensile tests on the rod material.

The pull-out energy (*W_P_*) and the specific pull-out energy (${\bar{W}}_{P}$) are key indices for evaluating the energy dissipation during the pull-out process of the pull-out material. The pull-out energy (*W_P_*) comprehensively reflects the energy dissipation over the whole process from the onset of loading to complete pull-out, expressed as:

(3) $$W_{P}=\int_{0}^{L_{E}}F\left(s\right)\mathrm{ds}$$

where *L_E_* denotes the anchorage length.

The specific pull-out energy is used to compare the energy dissipation efficiency per unit area under different bonded lengths, expressed as:

(4) $${\bar{W}}_{P}=\frac{W_{P}}{\pi D_{F}L_{E}}$$

The evolution of the interfacial shear stress between the bolt and the surrounding rock during pull-out can be evaluated by the average bond strength (*τ_av_*), which uniformly distributes the peak force over the entire anchorage interface and reflects the average shear stress level of the interface at the instant of failure, expressed as:

(5) $$\tau_{\mathrm{av}}=\frac{F_{\max}}{\pi D_{F}L_{E}}$$

The equivalent bond strength (*τ_eq_*) is back-calculated from an energy perspective, with the physical assumption that the interfacial shear stress is uniformly distributed along the anchorage length. The difference between the average bond strength (*τ_av_*) and the equivalent bond strength (*τ_eq_*) reflects the proportion of post-peak energy dissipation in the total pull-out energy. The mathematical expression of the equivalent bond strength (*τ_eq_*) is:

(6) $$\tau_{\mathrm{eq}}=\frac{2W_{P}}{\pi D_{F}{L_{E}}^{2}}$$

Based on 22 groups of bolt pull-out tests, this study analyzes the effects of different test variables (anchorage length, confining pressure, material strength, and expansion point type) on the interfacial anchorage performance between the bolt and the surrounding rock in similar materials. Among them, the conventional steel bolt similar material group comprises a total of 16 groups: the anchorage length group sets six anchorage lengths of 10 cm, 20 cm, 30 cm, 40 cm, 50 cm, and 60 cm to systematically study the influence of anchorage length on the interfacial anchorage performance, and the corresponding groups are denoted as S-A; the confining pressure group applies five levels of radial confinement stress of 0.10 MPa, 0.15 MPa, 0.20 MPa, 0.25 MPa, and 0.30 MPa to explore the effect of the confining pressure level on the interfacial confinement effect, and the corresponding groups are denoted as S-C; the material strength group adopts five types of surrounding rock similar materials with different strengths to analyze the regulatory role of surrounding rock strength on the interfacial anchorage performance, and the corresponding groups are denoted as S-M. It is noteworthy that the group with an anchorage length of 40 cm, M3 material strength, and 0.20 MPa confining pressure serves as the blank control group, providing a unified comparison baseline for the three test variables of anchorage length, confining pressure, and material strength. The expansion-type rock bolt similar material group comprises a total of 6 groups, in which expansion point structures with different numbers and sizes are set on the bolt surface to examine the enhancement effect of the expansion point structure on the interfacial performance, and the corresponding groups are denoted as E-N and E-S, respectively. The pull-out test results are grouped by specimen variables, with three replicates per group to ensure representativeness, and anomalous data are excluded (Table S2). The mean, standard deviation, and coefficient of variation of the four evaluation indices for all groups are summarized in Table S1. The statistical significance of the key comparisons was assessed by Welch’s *t*-tests (Table S3). The test results are shown in Table 2.

### 3.2. Anchorage Length Effect

**Figure 3:**
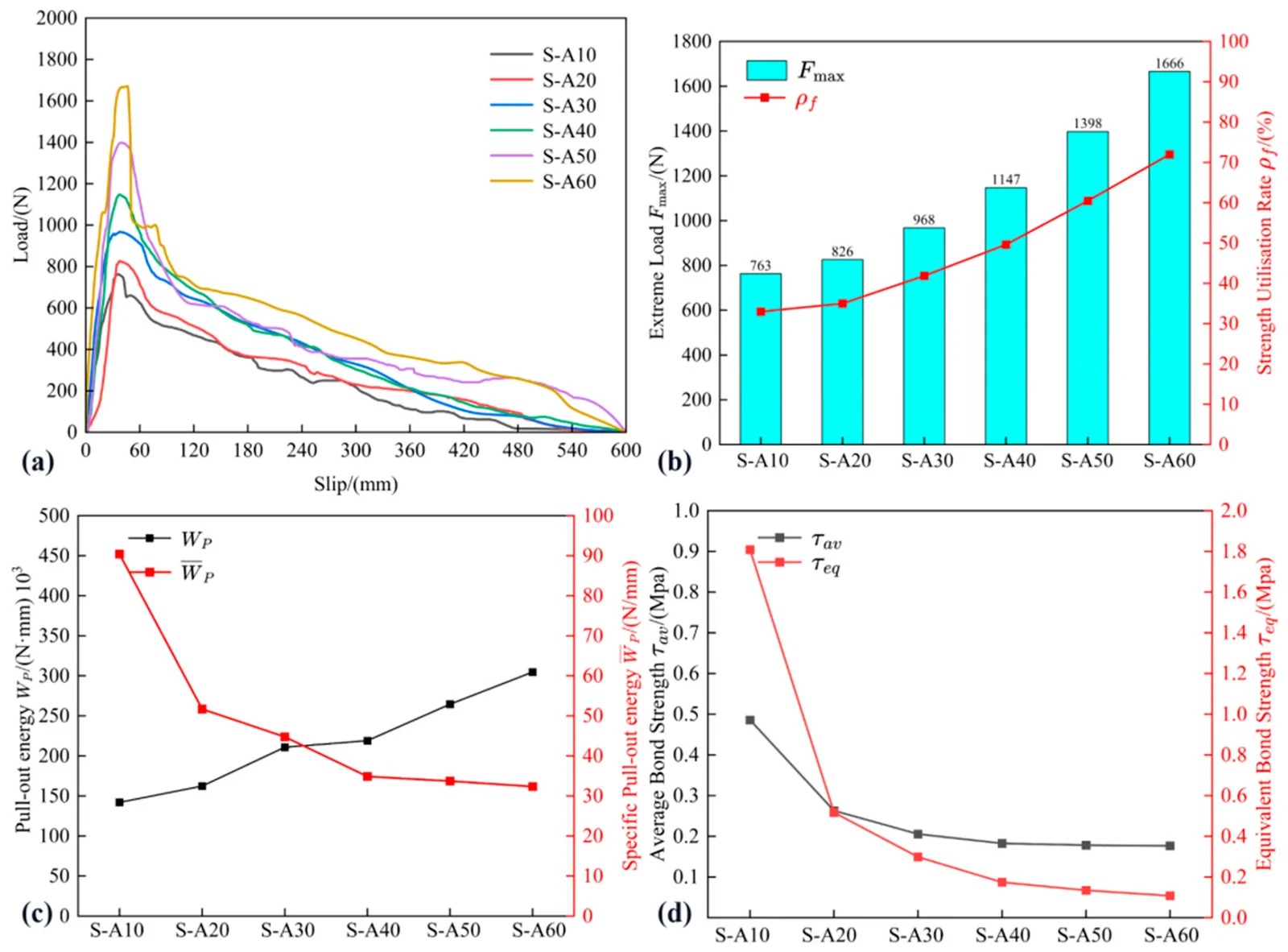
Load-slip curves and evaluation indices of steel bolts under different anchorage lengths: (**a**) load-slip curves; (**b**) the extreme load *F_max_* and the strength utilization ratio *ρ_f_*; (**c**) the pull-out energy *W_P_* and the specific pull-out energy ${\bar{W}}_{P}$; (**d**) the average bond strength *τ_av_* and the equivalent bond strength *τ_eq_*.

The anchorage length is the most fundamental controlling factor governing the anchorage effect. The load-slip curves and evaluation indices of steel bolts under different anchorage lengths are shown in Figure 3. As shown in Figure 3a, the ascending segments of the groups under different anchorage lengths all tend toward linear behavior, but there are relatively obvious differences in the descending segments after the peak. As the anchorage depth gradually increases, the peak load increases significantly; for example, compared with Group S-A10, the extreme load of Group S-A60 increases by 118%, and the residual stress during the pull-out process also gradually increases by approximately 109%. As the anchorage length increases stepwise from 10 cm to 60 cm, the extreme load increases by 8.3%, 17.2%, 18.5%, 21.9%, and 19.2%, respectively, indicating that increasing the anchorage length can effectively enhance the bonding at the bolt-surrounding rock interface, albeit with a marginal effect. Figure 3c shows that the pull-out energy increases with the anchorage length, while the specific pull-out energy decreases with the increase in anchorage length, indicating that short anchorages exhibit the highest stress concentration and the highest energy dissipation per unit bonded area. Correspondingly, after the peak, the residual load of short anchorages remains at a low level over a long slip distance, whereas long anchorages sustain a higher residual load over the same pull-out displacement. From Figure 3d, it can be seen that the average bond strength and equivalent bond strength are inversely proportional to the anchorage length; as the anchorage length increases, the interfacial contact area increases, while the pull-out ductility decreases instead. This decrease arises because the shear stress along a longer anchorage is mobilized non-uniformly: the bonded zone near the loaded end debonds progressively, so that the residual slip available after the peak does not grow in proportion to the anchorage length. The simultaneous decrease in the average and equivalent bond strengths with increasing anchorage length is consistent with this interpretation. When the anchorage length increases from 10 cm to 20 cm, the equivalent bond strength decreases significantly, representing the transition of the test group from short anchorage to long anchorage. Figure 3c,d jointly indicate that the marginal benefit of increasing the anchorage length decreases for longer anchorages, which should be taken into account in support design. The improvement of the anchorage performance with increasing anchorage length is consistent with a mechanism dominated by the enlarged bonded area, although a concurrent change in the failure mode cannot be fully excluded.

### 3.3. Confining Pressure Effect

**Figure 4:**
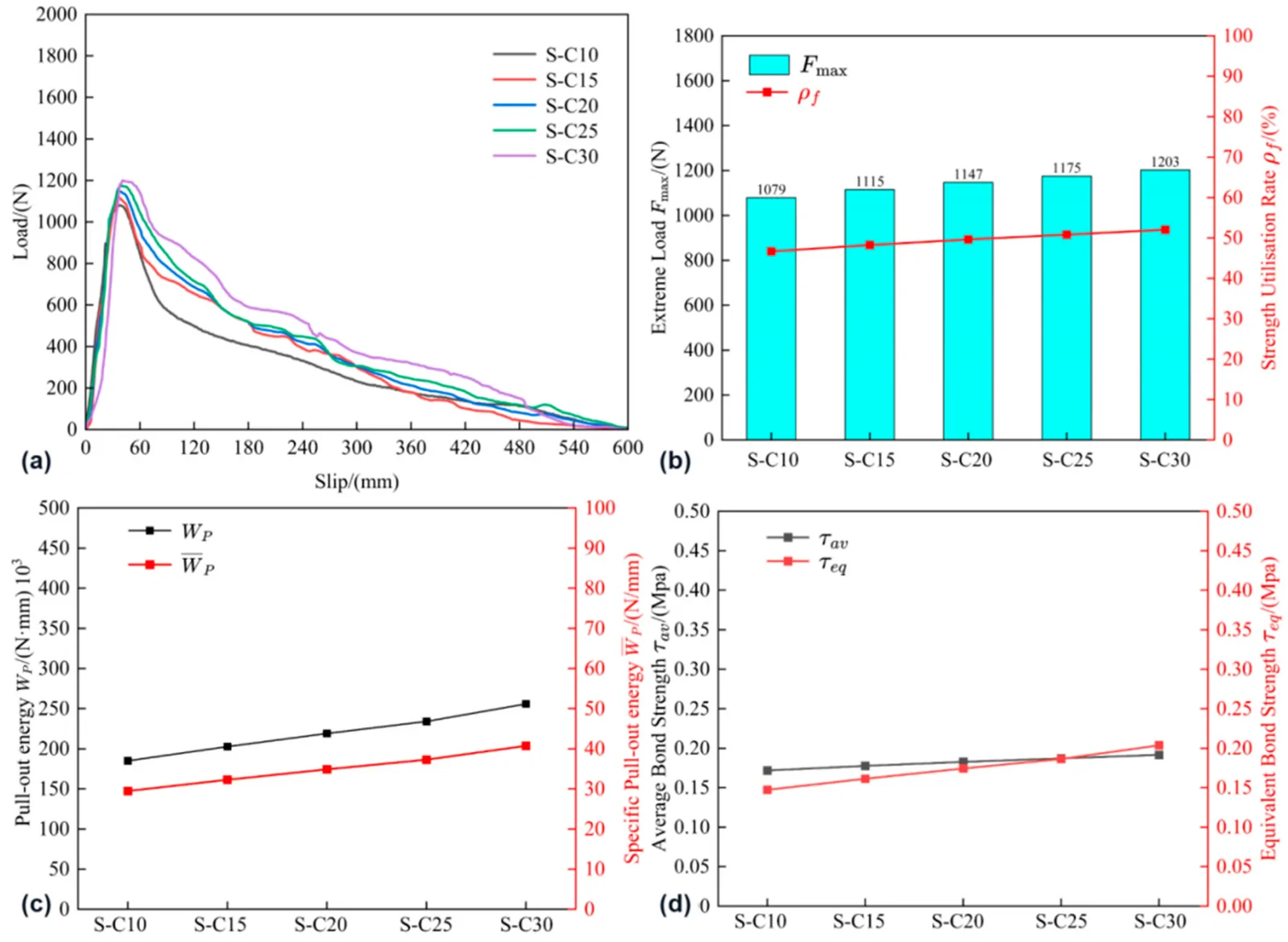
Load-slip curves and evaluation indices of steel bolts under different confining pressure conditions: (**a**) load-slip curves; (**b**) the extreme load *F_max_* and the strength utilization ratio *ρ_f_*; (**c**) the pull-out energy *W_P_* and the specific pull-out energy ${\bar{W}}_{P}$; (**d**) the average bond strength *τ_av_* and the equivalent bond strength *τ_eq_*.

By changing the confining pressure, the lateral confinement of the surrounding rock on the bolt under different burial depths can be explored, and its effect on the anchorage performance can be further analyzed. The load-slip curves and evaluation indices of steel bolts under different confining pressure conditions are shown in Figure 4. From Figure 4a,b, it can be seen that with the increase in confining pressure, the extreme load gradually increases, but compared with other test factors, the increase in the extreme load brought by the increase in confining pressure is not obvious. Compared with Group S-C10, the extreme load of Group S-C30 increases by only 11.5%, while the intermediate Groups S-C15, S-C20, and S-C25 show smaller increases of 3.3%, 6.3%, and 8.9%, respectively. On the one hand, this indicates that the increment of confining pressure converted into the increment of the interfacial radial confinement force has undergone a significant reduction through the interfacial friction coefficient; on the other hand, considering that the anchorage length of this group is uniformly 40 cm, it indicates that under a long anchorage system, the increase in confining pressure mainly acts on the distal end of the anchorage, while the improvement of the proximal anchorage region where the extreme load is located is limited. Because the pull-out load is transferred progressively from the loaded end towards the distal end, only the portion of the anchorage that is still bonded carries the load at a given moment. Under long anchorage, the proximal zone debonds first near the peak, so the additional radial confinement acts mainly on the distal zone during the post-peak stage; this explains why the peak load increases only slightly, whereas the post-peak resistance and energy dissipation increase markedly. Quantitatively, the residual load at a displacement of 300 mm increases from approximately 233 N at 0.10 MPa to approximately 370 N at 0.30 MPa, which directly quantifies the enhancement of the residual frictional resistance by the confining pressure. Figure 4c shows that the pull-out energy and the specific pull-out energy increase approximately linearly with the confining pressure. From the perspective of microscopic mechanisms, the confining pressure enhances the energy dissipation through the following pathways [39,40]: (1) increasing the interfacial friction coefficient; (2) suppressing the opening and propagation of bond cracks; and (3) improving the residual frictional bearing capacity of the fractured zone. The increments of the pull-out energy between successive groups are 9.5%, 8.14%, 6.86%, and 9.25%, respectively, which are relatively uniform, indicating that with the increase in confining pressure, the pull-out energy and specific pull-out energy increase almost linearly. Figure 4d shows that the increase in confining pressure promotes the improvement of the equivalent bond strength, but the variation in the average bond strength is relatively small. However, the ratio of the pull-out energy increment to the peak load increment is 3.5, the highest among all test factors, which, together with the contents of Figure 4c,d, further suggests that under long anchorage, the effect of confining pressure on the anchorage strength is more reflected in the post-peak stage.

### 3.4. Material Strength Effect

**Figure 5:**
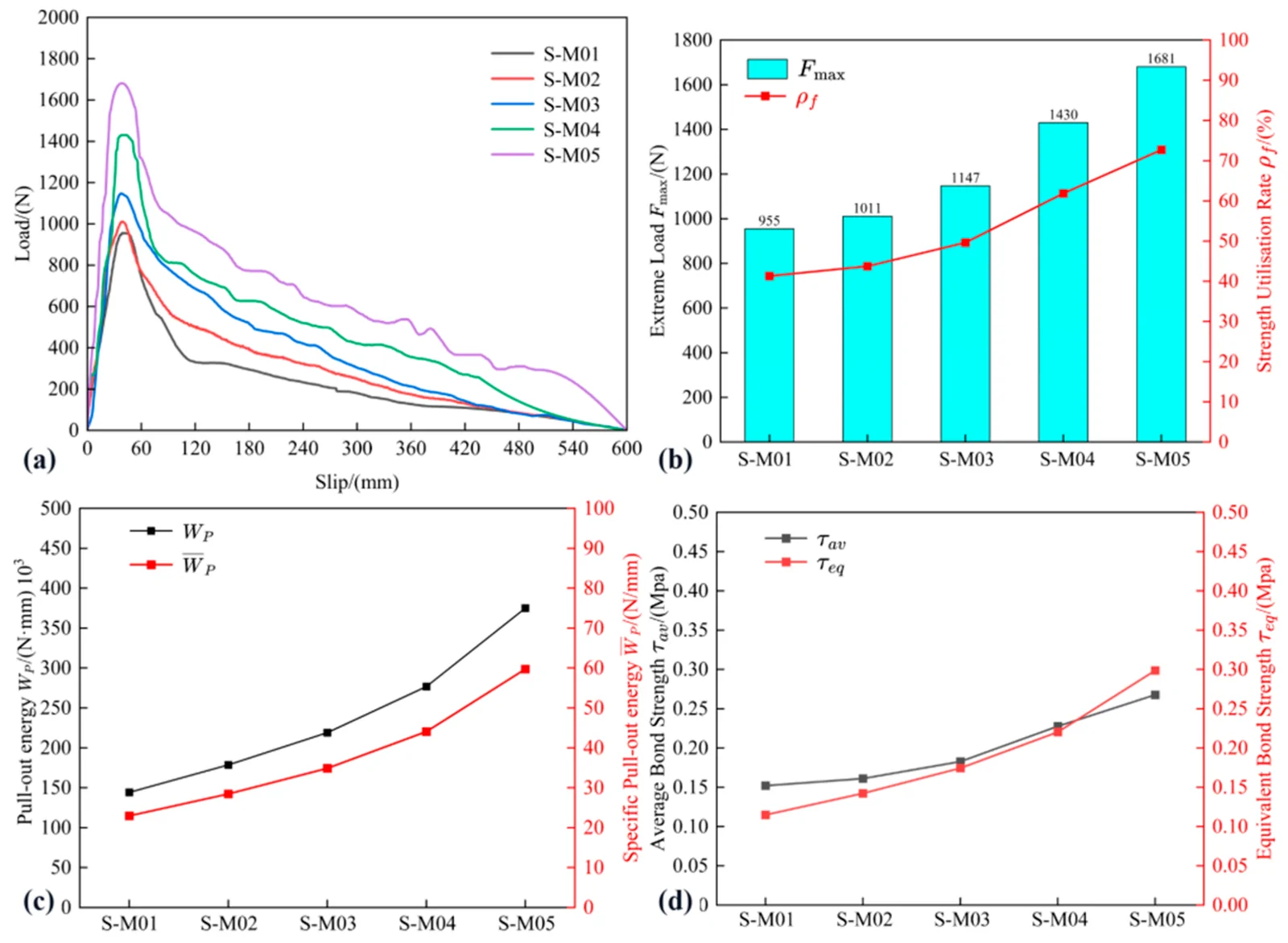
Load-slip curves and evaluation indices of steel bolts under different material strength: (**a**) load-slip curves; (**b**) the extreme load *F_max_* and the strength utilization ratio *ρ_f_*; (**c**) the pull-out energy *W_P_* and the specific pull-out energy ${\bar{W}}_{P}$; (**d**) the average bond strength *τ_av_* and the equivalent bond strength *τ_eq_*.

The surrounding rock strength is a key factor determining the failure mode. When the surrounding rock strength is much higher than the interfacial strength, slip failure occurs at the interface; when the surrounding rock strength decreases, the interface gradually turns to a mixed failure mode combining slip failure and cone failure. The load-slip curves and evaluation indices of steel bolts under different material strength are shown in Figure 5. From Figure 5a, it can be seen that the change in surrounding rock strength significantly improves the load-slip curves: in the ascending segment, the extreme load of Group S-M05 increases by 76% compared with Group S-M01, and the average residual force in the descending segment is about 3 times higher. From Figure 5c, it can be seen that the improvement of surrounding rock strength also effectively enhances the pull-out energy: compared with Group S-M01, the pull-out energy of Group S-M05 increases by 160%, and the ratio to the increment of the peak load is 2.1, further proving the finding revealed in Figure 5a that the change in surrounding rock strength significantly alters the load throughout the pull-out process. At the microscopic level, with the increase in material strength, the chemical bonds and mechanical interlocking at the bonding interface are enhanced, and the interfacial failure tends to shift from adhesive failure to cohesive failure within the matrix, as suggested by the post-test failure patterns of the specimens. These inferences are drawn from the measured load-slip responses: the enhanced bonding is indicated by the higher peak load and the steeper ascending branch; the higher energy required for crack propagation is indicated by the 160% increase in the pull-out energy, which exceeds the 76% increase in the peak load; and the higher residual friction of the fractured zone is consistent with the residual load of the descending segment, which is on average about three times higher for S-M05 than for S-M01.

### 3.5. Expansion Point Effect

The anchorage force of conventional bolts is mainly realized through the interfacial bonding of the anchorage section. On this basis, the expansion-type rock bolt forms expansion points through methods such as injecting the anchoring agent, transforming the interfacial load transfer from pure bonding to a composite mode in which multiple factors act together. The size of the formed expansion point is mainly determined by the grouting method. Relevant studies show that improving the grouting method and enhancing the grout strength can effectively increase the expansion point size [41,42]; therefore, this paper controls the expansion point size to equivalently represent the improvement of the grouting method. The number of expansion points affects the total expansion length on the bolt and the bolt shape, and is a key factor in studying the anchorage mechanism of the expansion-type rock bolt. Therefore, this test selects the expansion point size and the number of expansion points as factors to study the expansion-type rock bolt. The size, number, and positions of the expansion points for all groups are summarized in Table 3.

**Figure 6:**
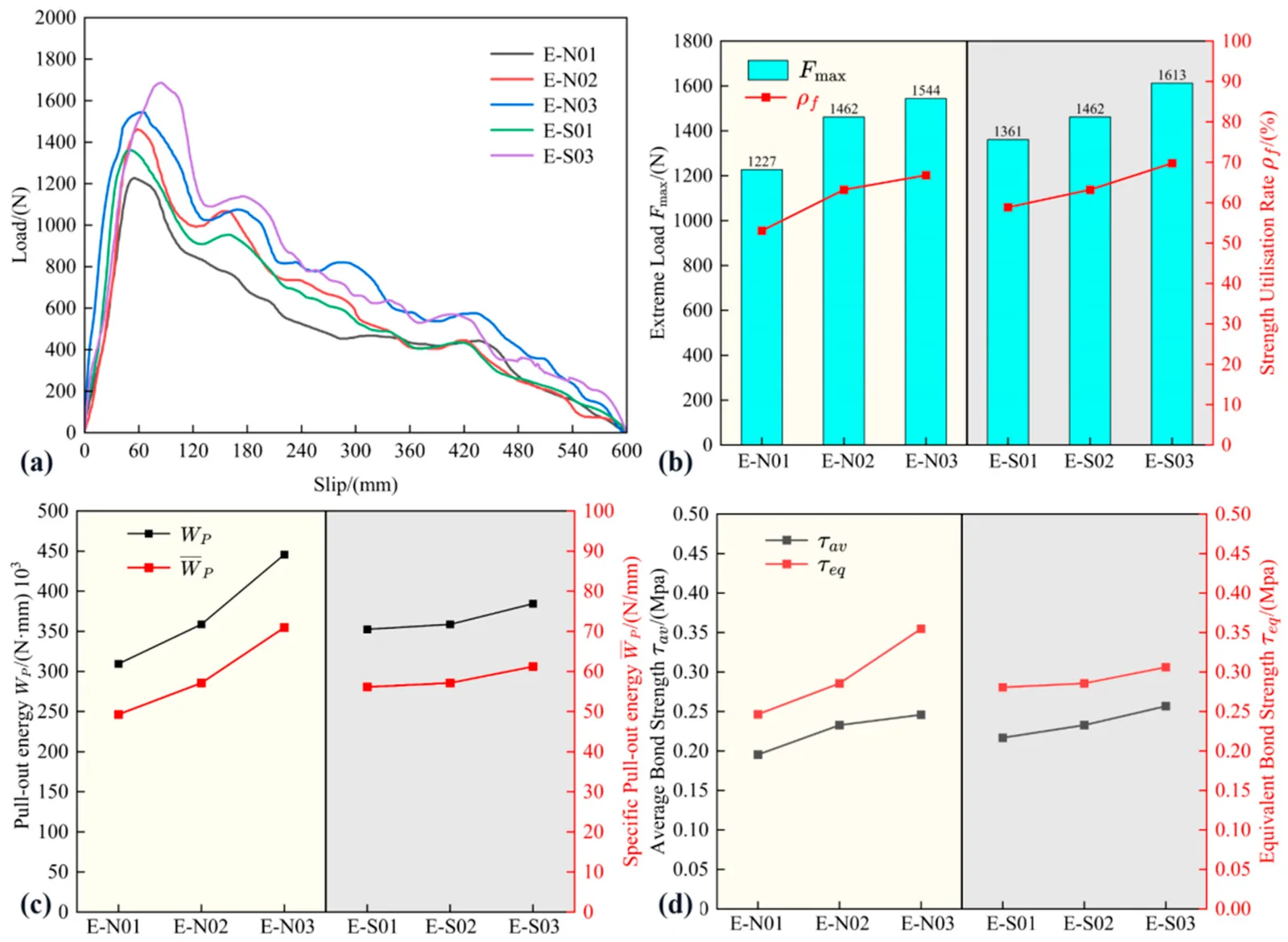
Load-slip curves and evaluation indices of expansion-type rock bolts under different expansion point sizes and numbers of expansion points: (**a**) load-slip curves; (**b**) the extreme load *F_max_* and the strength utilization ratio *ρ_f_*; (**c**) the pull-out energy *W_P_* and the specific pull-out energy ${\bar{W}}_{P}$; (**d**) the average bond strength *τ_av_* and the equivalent bond strength *τ_eq_*.

The load-slip curves and evaluation indices of expansion-type rock bolts under different expansion point sizes and numbers of expansion points are shown in Figure 6. Note that E-S02 and E-N02 are the same test group, which is denoted as E-S02 in the figure. From Figure 6a, it can be seen that compared with steel bolts, the groups of expansion-type rock bolts show relatively obvious fluctuations in the descending segment, and the descending segments are generally gentler. From Figure 6b, it can be seen that both the increase in the number of expansion points and the increase in the expansion point size promote the enhancement of the anchorage force: the peak load of E-N02 increases by 19.2% compared with E-N01, while the peak load of E-N03 increases by only 5.6% compared with E-N02, with a significantly reduced increment, indicating a marginal effect of the number of expansion points. From Figure 6c, it can be seen that the increase in the expansion point size and the increase in the number of expansion points both effectively enhance the pull-out energy and the specific pull-out energy, among which the increase in the number of expansion points enhances the pull-out energy more significantly: compared with E-N01, the pull-out energy of E-N02 increases by 15.9%, and that of E-N03 increases by 24.2% compared with E-N02. However, with the increase in the expansion point size, the increment of the pull-out energy is lower than the increment of the extreme load. Figure 6b,c indicate that when the number of expansion points is larger, the anchorage force is dispersed over a longer anchorage section, contributing more to the energy dissipation of the continuous post-peak pull-out process, while the expansion point size directly contributes to the peak bearing capacity by providing a larger single-point anchorage contact area and a higher single-point anchorage force.

From Figure 6d, it can be seen that the increases in the expansion point size and the number of expansion points both improve the average bond strength and the equivalent bond strength, but in terms of the increments, the improvement by the expansion point size is relatively uniform while that by the number of expansion points is non-uniform. Compared with E-N01, the average bond strength and equivalent bond strength of E-N02 increase by 19.5% and 15.9%, respectively; compared with E-N02, those of E-N03 increase by 5.6% and 24.2%, respectively—the increment of the average bond strength decreases substantially while that of the equivalent bond strength increases substantially. Compared with E-S01, the average bond strength and equivalent bond strength of E-S02 increase by 7.4% and 1.8%, respectively; compared with E-S02, those of E-S03 increase by 10.3% and 7.2%, respectively. Assuming that each expansion point provides one anchorage reinforcement point during the pull-out process: when there is only one expansion point, the interfacial stress is concentrated at that point, and as the expansion point is damaged, the interfacial friction decreases rapidly. With the increase in the number of expansion points, multiple expansion points are distributed longitudinally along the bolt and are damaged one by one, effectively enhancing the load after the peak; while the change in the expansion point size does not change the longitudinal stress distribution of the bolt, and its effect is more reflected in increasing the extreme load by enlarging the pull-out cross-sectional area.

## 4. Discussion

### 4.1. Comparison of Pull-Out Stages

To further study the differences between conventional bolts and expansion-type rock bolts in the pull-out process, the basic control groups of the conventional bolt group and the expansion-type rock bolt group are selected for a detailed analysis of their pull-out stages.

**Figure 7:**
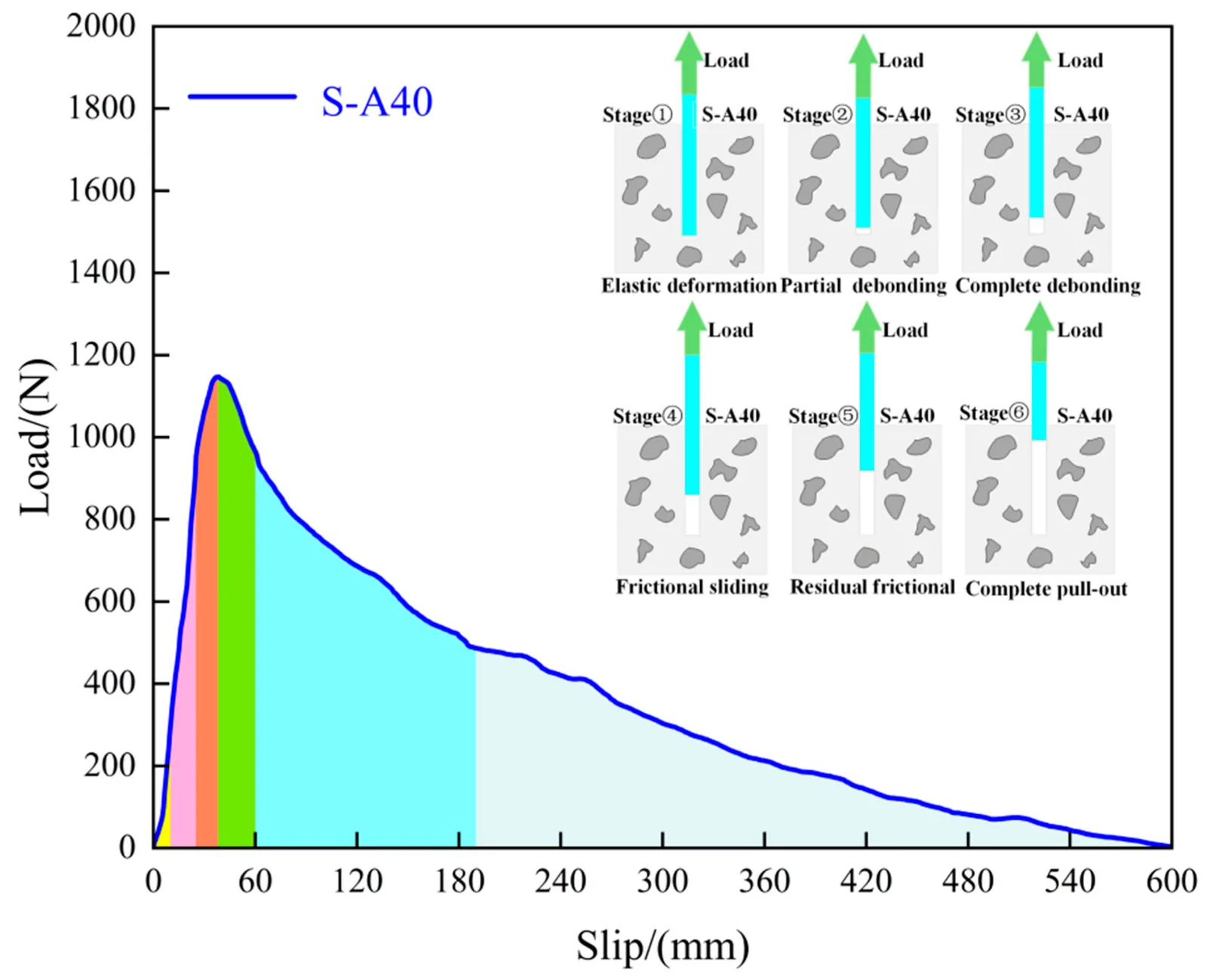
Load-slip curve and pull-out stage schematic of a conventional bolt.

The load-slip curve and pull-out stages of the conventional bolt are shown in Figure 7. The pull-out process of the conventional bolt is mainly divided into six stages. (1) The initial fully bonded stage exhibits a linear load-slip relationship, in which the bolt and the matrix maintain a fully bonded state through chemical adhesion, and the interface is free of any damage; with the further increase in the load, the bolt undergoes elastic elongation under the condition of intact bonding. The above stages are collectively referred to as the elastic deformation stage. This stage is shown in yellow in the figure. (2) In the subsequent partial debonding stage, bond degradation begins, the load increases rapidly at first and then slowly, accompanied by local slip and the loss of chemical bonding. This stage is shown in pink in the figure. (3) When the load gradually rises to the peak, the debonding front has advanced to the end of the anchorage section, with only a small fraction of the bonded area remaining, and the load is almost entirely concentrated in a very small region at the end; this is referred to as the complete debonding stage. This stage is shown in orange in the figure. (4) After complete bond failure, the bolt enters full-length slip. At this time, the load curve drops rapidly because the instantaneous release of elastic stored energy upon bond failure causes the stress to drop, while the interfacial asperities in the initial slip stage have not yet formed stable frictional contact, and the friction coefficient is at its minimum; as the pull-out process continues, the rate of force decay becomes slightly slower. The above stages are collectively referred to as the frictional sliding stage. This stage is shown in green in the figure. (5) Afterwards, the curve enters the residual frictional stage, which is characterized by an extremely slow decrease in the force with increasing displacement, and the load at this stage almost completely follows Coulomb’s friction law. This stage is shown in blue in the figure. (6) Finally, as the pulled-out length reaches the embedment length, the bolt is completely pulled out and the load returns to zero; this is referred to as the complete pull-out stage. This stage is shown in grey in the figure.

**Figure 8:**
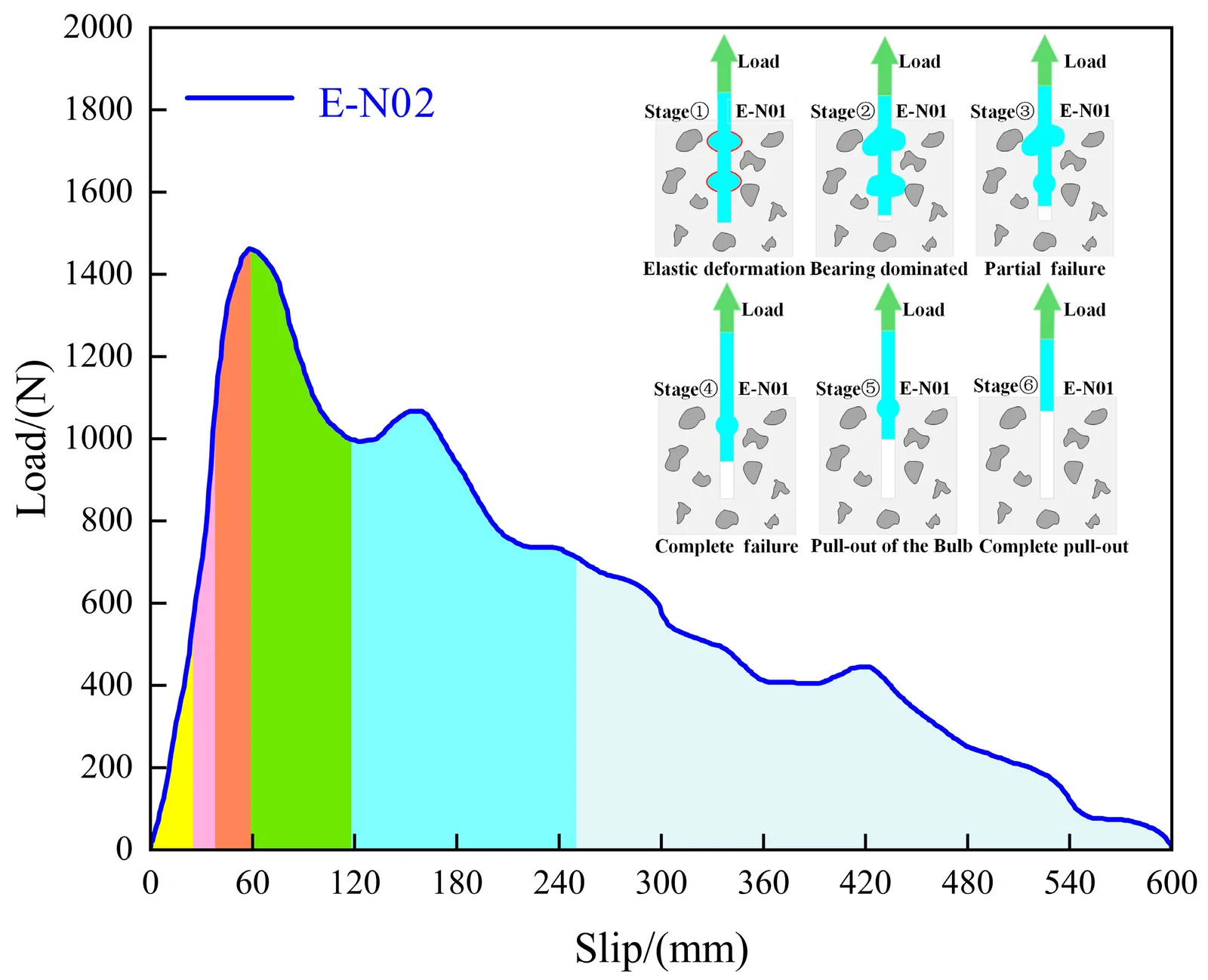
Load-slip curve and pull-out stage schematic of an expansion-type rock bolt.

The load-slip curve and pull-out stages of the expansion-type rock bolt are shown in Figure 8. The overall pull-out stages of the expansion-type rock bolt are similar to those of the conventional bolt, but the behavior of some stages differs significantly. (1) In the elastic deformation stage, the expansion-type rock bolt and the conventional bolt are consistent in the law of load increase; because the formation of the expansion points increases the local cross-sectional area of the bolt and improves the axial stiffness, the growth rate of the expansion-type rock bolt in this stage is slightly faster. This stage is shown in yellow in the figure. (2) In the subsequent partial debonding stage, the expansion points effectively provide compressive stress perpendicular to the pull-out direction, mobilizing additional frictional resistance, which effectively resists the trend of the interfacial chemical adhesion being gradually weakened as the debonding expands. This stage is shown in pink in the figure. (3) In the complete debonding stage, since the interfacial chemical adhesion has completely failed, the load is borne by the mechanical interlocking force provided by the system composed of multiple expansion points acting jointly; as the pull-out process continues, the expansion points are damaged one after another, and the bolt enters the slip stage. This stage is shown in orange in the figure. (4) In the frictional sliding stage, because the damaged expansion points still partially remain on the bolt body, the interfacial friction is effectively increased; therefore, the load in this stage is significantly greater than that of the conventional bolt. At a pull-out position of about 200 mm, which corresponds to the location of the first expansion point from the borehole mouth, the residual part of the first expansion point is pulled out, causing a rapid rise in the load and forming a small peak with a magnitude of approximately 1066 N, about 73% of the main peak. This stage is shown in green in the figure. (5) In the residual frictional stage, the load of the expansion-type rock bolt group decreases slowly with fluctuations; after the residual section of the second expansion point is pulled out, the decline rate of the load becomes faster, and at this time the load-slip curve is similar to that of the conventional bolt group. This stage is shown in blue in the figure. (6) Finally, as the pulled-out length reaches the embedment length, the bolt is completely pulled out and the load returns to zero. This stage is shown in grey in the figure.

### 4.2. Analysis of Bolt Deformation and Failure

To further analyze the deformation and failure characteristics of the bolt after pull-out, diameter measurements were carried out at the head of some bolts after the test using a vernier caliper. The diameter of each bolt was measured in three different directions at the same position and averaged to avoid the influence of measurement errors. The bolt head diameters and corresponding necking ratios of some groups after pull-out are shown in Table 4. From the table, it can be seen that with the increase in anchorage length, the necking ratio of the bolt increases significantly: the necking ratio of Group S-A60 reaches 9.4%. Combined with the analysis in Section 3.2, the extreme load of this group is 1666 N, and the corresponding cross-sectional tensile stress reaches 84.9 MPa. The 1050 aluminium rod has a manufacturer-specified nominal yield strength of greater than 20 MPa, and its ultimate tensile strength was determined as approximately 117.8 MPa by uniaxial tensile tests on the rod material. The stress of 84.9 MPa therefore exceeds the nominal yield strength by more than a factor of four and reaches approximately 72% of the ultimate tensile strength, indicating entry into a significant strain-hardening stage. Accounting for the reduced cross-sectional area after necking, the true stress is approximately 93.7 MPa and the actual strength utilization ratio is approximately 79.5%. The bolt of this group therefore approached the verge of fracture.

Different from the anchorage length groups, the necking ratios of Groups E-N02 and E-N01 are the same, and the necking ratio of Group E-N03 decreases slightly compared with Group E-N02. With the increase in the number of expansion points, the necking ratio shows an overall change characteristic of remaining constant and then decreasing slightly. This indicates that the increase in the number of expansion points may help to disperse the load accumulation path, thereby protecting the bolt body and contributing to the further realization of the anchorage efficiency.

Compared with their respective control groups, the relative increases in the extreme load are up to 118% for the anchorage length between S-A10 and S-A60, 11.5% for the confining pressure between S-C10 and S-C30, 76% for the material strength between S-M01 and S-M05, 25.8% for the number of expansion points between E-N01 and E-N03, and 18.5% for the expansion-point size between E-S01 and E-S03. The anchorage length thus exerts the strongest effect on the peak load, whereas the confining pressure exerts the weakest peak effect but the strongest post-peak effect, with an energy-increment ratio of 3.5, consistent with the different governing mechanisms discussed above.

### 4.3. Limitations

Several interpretations in this study (e.g., the microscopic mechanisms of the confining-pressure effect and the load-dispersion mechanism of the expansion points) are inferred from the measured load-slip responses and the post-test observations rather than directly measured. Distributed strain measurements, acoustic-emission monitoring, and imaging of the interface would provide more direct evidence and are planned for future work. The results obtained at the 1:5 model scale should also be regarded as qualitative guidance for full-scale design, because scale effects (stress gradients and particle-size effects) cannot be fully reproduced.

## 5. Conclusions

Through similar bolt pull-out tests, the anchorage performance of the bolt similar material–surrounding rock similar material anchorage interface during pull-out was systematically studied under various test factors. A multi-dimensional evaluation was carried out combined with multiple indices, and the interfacial failure mechanism was further analyzed in conjunction with the pull-out stages and the bolt deformation. Based on the results of this study, the main conclusions are drawn as follows:

(1) The anchorage length improves both the bearing capacity and the energy dissipation mainly by enlarging the bonded area; the confining pressure enhances the post-peak interfacial frictional behaviour far more than the peak bearing capacity; and the material strength improves both the peak force and the pull-out energy most comprehensively. The corresponding results are consistent with mechanisms associated with the geometric scale, the interfacial confinement, and the matrix properties, respectively.
(2) The expansion-type rock bolt changes the interfacial load transfer path by forming expansion points on the bolt surface, achieving non-proportional enhancement of the bearing capacity and energy dissipation. The increase in the number of expansion points helps to disperse the load accumulation path, while the increase in the expansion point size significantly enhances the peak load mainly by improving the bearing capacity of a single expansion point.
(3) The pull-out process consists of six consecutive stages—the elastic deformation stage, partial debonding stage, complete debonding stage, frictional sliding stage, residual frictional stage, and complete pull-out stage—and the expansion-type rock bolt shows relatively obvious differences from the conventional bolt in the above pull-out stages. This six-stage description is valid for the configurations tested in this study and is intended as an interpretation of the measured responses rather than a universal pull-out mechanism.

## Figures and Tables

**Table 1:** Material mix proportions.

Group	Quartz Sand 20–40 Mesh	Quartz Sand 40–100 Mesh	Quartz Sand 100–200 Mesh	Quartz Sand 300–400 Mesh	Building Gypsum	Water	Cement C30
S-M01	10.48 kg	26.21 kg	10.48 kg	5.24 kg	7.86 kg	4.95 L	0
S-M02	9.95 kg	24.88 kg	9.95 kg	4.98 kg	9.95 kg	5.97 L	1.67 kg
S-M03	9.47 kg	23.69 kg	9.47 kg	4.74 kg	11.84 kg	6.75 L	3.47 kg
S-M04	9.04 kg	22.60 kg	9.04 kg	4.52 kg	13.56 kg	7.19 L	5.35 kg
S-M05	8.64 kg	21.61 kg	8.64 kg	4.32 kg	15.13 kg	7.56 L	7.32 kg

**Table 2:** Pull-out test results.

Test No.	*F_max_*/N	*W_P_*/(N·mm) (10^3^)	${\bar{W}}_{P}$ (N/mm)	*σ_f_*_,*max*_/MPa	*τ_av_*/MPa	*τ_eq_*/MPa	*ρ_f_*/%
S-A10	763	141.93	90.40	38.88	0.49	1.81	33
S-A20	826	162.35	51.70	42.09	0.26	0.52	36
S-A30	968	210.85	44.77	49.32	0.21	0.30	42
S-A40	1147	219.07	34.88	58.45	0.18	0.17	50
S-A50	1398	264.52	33.70	71.24	0.18	0.13	60
S-A60	1666	304.76	32.35	84.89	0.18	0.11	72
S-C10	1079	185.00	29.46	54.98	0.17	0.15	47
S-C15	1115	202.58	32.26	56.82	0.18	0.16	48
S-C20	1147	219.07	34.88	58.45	0.18	0.17	50
S-C25	1175	234.09	37.28	59.87	0.19	0.19	51
S-C30	1203	255.75	40.72	61.30	0.19	0.20	52
S-M01	955	144.18	22.96	48.66	0.15	0.11	41
S-M02	1011	178.63	28.44	51.52	0.16	0.14	44
S-M03	1147	219.07	34.88	58.45	0.18	0.17	50
S-M04	1430	276.69	44.06	72.87	0.23	0.22	62
S-M05	1681	375.02	59.72	85.66	0.27	0.30	73
E-N01	1227	309.55	49.29	62.52	0.20	0.25	53
E-N02	1462	358.69	57.12	74.50	0.23	0.29	63
E-N03	1544	445.64	70.96	78.68	0.25	0.35	67
E-S01	1361	352.50	56.13	69.35	0.22	0.28	59
E-S02	1462	358.69	57.12	74.50	0.23	0.29	63
E-S03	1613	384.50	61.23	82.19	0.26	0.31	70

**Table 3:** Size, number, and positions of the expansion points for the different test groups.

Group	Expansion Point Size	Expansion Point Width/mm	Expansion Point Length/mm	Number of Expansion Points	Distance from the Bolt Head to the Center of Expansion Point 1/mm	Distance from the Bolt Head to the Center of Expansion Point 2/mm	Distance from the Bolt Head to the Center of Expansion Point 3/mm
E-S01	Small	10	40	2	200	400	–
E-S02	Middle	14	60	2	200	400	–
E-S03	Large	18	80	2	200	400	–
E-N01	Middle	14	60	1	200	–	–
E-N02	Middle	14	60	2	200	400	–
E-N03	Middle	14	60	3	200	400	570

**Table 4:** Average bolt head diameter and necking ratio after pull-out.

Group	Diameter 1/mm	Diameter 2/mm	Diameter 3/mm	Mean Diameter/mm	Necking Ratio/%
S-A10	5	4.9	4.9	4.93	2.8
S-A40	4.8	4.8	4.7	4.77	9
S-A60	5	4.8	4.5	4.76	9.4
E-N01	4.7	4.7	4.9	4.77	9
E-N02	4.7	4.8	4.8	4.77	9
E-N03	4.8	4.7	4.9	4.8	7.8

## Data Availability

The original contributions presented in this study are included in the article/Supplementary Materials. Further inquiries can be directed to the corresponding author.

## Supplementary Materials

The following supporting information can be downloaded at: https://www.mdpi.com/article/10.3390/ma19204264/s1, Table S1: Mean, standard deviation, and coefficient of variation of the four evaluation indices for all test groups; Table S2: Excluded specimens and the corresponding reasons; Table S3: Welch’s *t*-tests for the key comparisons.

## Abbreviations

The following abbreviations are used in this manuscript:

AE	acoustic emission
DIC	digital image correlation
FBG	fiber Bragg grating
