# Supplementary material for: Analysis of Interfacial Properties and Mechanical Mechanisms of Expansion-Type Bolts
Source: Materials (Basel). 2026 Oct 9;19(20):4264. doi: 10.3390/ma19204264 (PMC13707010; doi:10.3390/ma19204264)
Supplement: Supplementary file 1 [file materials-19-04264-s001.zip › materials-4556199-supplementary.pdf]

Supplementary Materials

The information presented below supports the analyses reported in the main text; because it is not the focus of the discussion in the paper, it is provided here as supplementary material.

Table S1. Mean, standard deviation, and coefficient of variation of the four evaluation indices for all test groups

Group	F_{max}/N	CoV	W_P/(N·mm)	CoV	τ_{av}/MPa	CoV	τ_{eq}/MPa	CoV
S-A10	763 ± 54.94	7.2%	141,930 ± 13,909.14	9.8%	0.49 ± 0.035	7.2%	1.81 ± 0.177	9.8%
S-A20	826 ± 49.56	6.0%	162,350 ± 13,799.75	8.5%	0.26 ± 0.016	6.0%	0.52 ± 0.044	8.5%
S-A30	968 ± 52.27	5.4%	210,850 ± 16,446.30	7.8%	0.21 ± 0.011	5.4%	0.3 ± 0.023	7.8%
S-A40	1147 ± 57.35	5.0%	219,070 ± 15,773.04	7.2%	0.18 ± 0.009	5.0%	0.17 ± 0.012	7.2%
S-A50	1398 ± 67.1	4.8%	264,520 ± 18,516.40	7.0%	0.18 ± 0.009	4.8%	0.13 ± 0.009	7.0%
S-A60	1666 ± 86.63	5.2%	304,760 ± 20,723.68	6.8%	0.18 ± 0.009	5.2%	0.11 ± 0.007	6.8%
S-C10	1079 ± 56.11	5.2%	185,000 ± 17,575.00	9.5%	0.17 ± 0.009	5.2%	0.15 ± 0.014	9.5%
S-C15	1115 ± 56.87	5.1%	202,580 ± 16,611.56	8.2%	0.18 ± 0.009	5.1%	0.16 ± 0.013	8.2%
S-C20	1147 ± 57.35	5.0%	219,070 ± 15,773.04	7.2%	0.18 ± 0.009	5.0%	0.17 ± 0.012	7.2%
S-C25	1175 ± 56.4	4.8%	234,090 ± 15,918.12	6.8%	0.19 ± 0.009	4.8%	0.19 ± 0.013	6.8%
S-C30	1203 ± 55.34	4.6%	255,750 ± 16,623.75	6.5%	0.19 ± 0.009	4.6%	0.2 ± 0.013	6.5%
S-M01	955 ± 74.49	7.8%	144,180 ± 14,706.36	10.2%	0.15 ± 0.012	7.8%	0.11 ± 0.011	10.2%
S-M02	1011 ± 64.7	6.4%	178,630 ± 15,362.18	8.6%	0.16 ± 0.01	6.4%	0.14 ± 0.012	8.6%
S-M03	1147 ± 57.35	5.0%	219,070 ± 15,773.04	7.2%	0.18 ± 0.009	5.0%	0.17 ± 0.012	7.2%
S-M04	1430 ± 62.92	4.4%	276,690 ± 16,601.40	6.0%	0.23 ± 0.01	4.4%	0.22 ± 0.013	6.0%
S-M05	1681 ± 67.24	4.0%	375,020 ± 19,501.04	5.2%	0.27 ± 0.011	4.0%	0.3 ± 0.016	5.2%
E-N01	1227 ±	6.2%	309,550 ±	8.4%	0.2 ±	6.2%	0.25 ±	8.4%

	76.07		26,002.20		0.012		0.021	
E-N02	1462 ± 84.8	5.8%	358,690 ± 28,695.20	8.0%	0.23 ± 0.013	5.8%	0.29 ± 0.023	8.0%
E-N03	1544 ± 92.64	6.0%	445,640 ± 39,216.32	8.8%	0.25 ± 0.015	6.0%	0.35 ± 0.031	8.8%
E-S01	1361 ± 88.47	6.5%	352,500 ± 28,905.00	8.2%	0.22 ± 0.014	6.5%	0.28 ± 0.023	8.2%
E-S02	1462 ± 84.8	5.8%	358,690 ± 28,695.20	8.0%	0.23 ± 0.013	5.8%	0.29 ± 0.023	8.0%
E-S03	1613 ± 88.72	5.5%	384,500 ± 29,991.00	7.8%	0.26 ± 0.014	5.5%	0.31 ± 0.024	7.8%

Table S2. Excluded specimens and the corresponding reasons

**Group**	**Specimen No.**	**Reason (EN)**
S-A20	3	measured peak load far below the parallel specimens
S-A60	1	sudden displacement-sensor anomaly with loud noise during loading
S-C10	1	measured peak load far below the parallel specimens
S-M04	1	the exposed bolt section was bent by an external impact during testing
E-N03	2	bolt fracture during loading
E-S03	3	bolt fracture during loading

Table S3. Welch's t-tests for the key comparisons

**Factor**	**Δ (N)**	**SE (N)**	**t**	**df**	**p**	**Conclusion**
Anchorage length	903	59.2	15.25	3.4	< 0.001	highly significant
Material strength	726	57.9	12.53	4.0	< 0.001	highly significant
No. of expansion points	317	69.2	4.58	3.9	≈ 0.011	significant
Expansion-point	252	72.3	3.48	4.0	≈ 0.025	significant

size						
Confining pressure	124	45.5	2.73	4.0	≈ 0.05	marginally significant
2nd expansion point	235	65.8	3.57	3.9	≈ 0.023	significant
3rd expansion point	82	72.5	1.13	3.9	≈ 0.32	not significant
